# Management of a Patient with Severe Osteogenesis Imperfecta Undergoing Laparoscopic Cholecystectomy

**DOI:** 10.1007/s00223-025-01375-9

**Published:** 2025-05-08

**Authors:** Sarah Scott, Helen Jordan, Laura Gill, Andreas Luhmann, Jamie Abbott, Stuart H. Ralston

**Affiliations:** 1 Department of Anaesthesia and Intensive Care, Victoria Hospital, Kirkcaldy, KY2 5AH UK; 2 Department of General Surgery, Victoria Hospital, Kirkcaldy, KY2 5AH UK; 3 Brittle Bone Society, 30 Guthrie St, Dundee, DD1 5BS UK; 4 Centre for Genomic and Experimental Medicine, Institute of Genetics and Cancer, University of Edinburgh, Western General Hospital, Edinburgh, EH4 2XU UK

**Keywords:** Osteogenesis imperfecta, Laparoscopic surgery, Anasthesia

## Abstract

Individuals with severe osteogenesis imperfecta who require surgery often present a difficult management problem due to limb deformity and shortening, kyphoscoliosis, and deformity of the rib cage. All of these features may be associated with respiratory problems and impaired cardiovascular reserve. Surgical procedures and anaesthetic management represent a substantial challenge in these individuals. Here, we describe the clinical outcome of laparoscopic surgery to remove multiple gallstones in an individual with severe osteogenesis imperfecta. Meticulous pre-operative planning, combined with careful anaesthetic management resulted in a favourable outcome with a beneficial effect on quality of life. We provide a detailed account of the challenges faced and how these were surmounted in the hope that this may be of benefit to other clinicians faced with similar problems. Our experience demonstrates that laparoscopic surgery can be successfully performed in people with severe osteogenesis imperfecta with a favourable outcome.

## Introduction

Osteogenesis imperfecta, or brittle bone disease, is a rare genetic disorder affecting one in 15–20,000 births. It is a multi-system disease affecting type 1 collagen; the most abundant protein in skin, bone, and tendon, which manifests as bone fragility and fractures as well as abnormalities in many other organ systems [1]. Osteogenesis imperfecta can arise as the result of mutations in 22 different genes but in about 80%, the disease occurs as the result of mutations in the *COL1A1* or *COL1A2* genes which encode the alpha 1 (I) and alpha 1 (2) protein chains, respectively [2]. The Sillence classification originally devised in 1979 is still used but only applies to disease severity in people with *COLIA1* or *COL1A2* mutations [3]. This case report concerns a patient with type III (progressively deforming) OI which invariably presents in infancy with multiple low trauma fractures that continue throughout life. These result in long bone deformity, chest deformity, and kyphoscoliosis. Affected patients are usually non-ambulatory and require assistive devices, such as motorised wheelchairs for mobility. Non-skeletal complications of OI include pulmonary disease, cardiovascular disease and gastrointestinal disease [1, 4]. There are few case reports of adult patients with type III OI successfully undergoing surgery, particularly non-orthopaedic surgery, due to the risk of complications in those with compromised cardio-respiratory function. This case report explores some considerations relating to both anaesthetic technique and surgical approach in an adult patient with severe OI who required a cholecystectomy because of multiple gallstones.

## Case Report

**Fig. 1:**
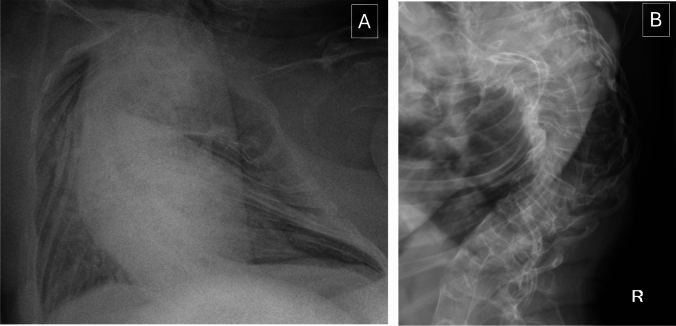
Radiographs of spine and chest. Panel A shows a chest X-ray prior to surgery demonstrating the pre-existing chest deformity. Panel B shows a lateral X-ray of the thoracic and upper lumbar spine demonstrating kyphoscoliosis

The patient is a 37-year-old man with type III OI caused by a c.4248 + 2T > C nucleotide change in *COL1A1* which is predicted to create an alternative acceptor splice site resulting in production of an abnormal type 1 (alpha 1) collagen chain. He is of short stature (estimated height 96.5cm and weight 27.8kg) is wheelchair bound and requires assistance to transfer and with activities of daily living. Musculoskeletal manifestations of OI include severe kyphoscoliosis, chest deformity (Fig. 1) and shortening of all limbs due to multiple previous fractures. He had previously been treated with multiple courses of intravenous bisphosphonates, but this treatment had been paused over the years prior to surgery due to concerns about slow healing of fractures.

The presentation was with intermittent episodes of abdominal pain, which were initially episodic but eventually were occurring after every meal. He was hospitalised one year prior to surgery with severe abdominal pain as the result of biliary colic. Investigations by ultrasound and MRI scan confirmed the presence of multiple gallstones. The decision to proceed with surgery was made following discussions between all members of the care team and with the patient who felt that on balance, the potential benefits of the procedure would outweigh the risks in view of the considerable negative effect the symptoms were having on his quality of life.

He attended on the day of surgery, following extensive pre-operative assessment, for a laparoscopic cholecystectomy. On the day of surgery, he had bilateral lower limb plaster casts treating recent tibial/fibular fractures. Pulmonary function tests showed severe restrictive lung disease (FVC 0.62L FEV-1 0.6L FEV-1/FVC% 95.95%) with resting peripheral oxygen saturation (SpO_2_) of 96% on air and desaturation to 85% on exertion. He was on established non-invasive bilevel positive airway pressure (BiPAP) ventilation with supplemental oxygen (0.5L) overnight at home. His ECG showed a sinus tachycardia, whilst an echocardiogram showed normal cardiac size and good function of both the left and right ventricles. His airway examination revealed a Mallampati score of 3 but otherwise he had a good range of neck movement, mouth opening, thyromental distance, and subluxation of his jaw.

Prior to induction of anaesthesia, meticulous attention was given to patient positioning with use of a pressure-relieving gel mattress and careful padding and support of pressure points to minimise the risk of skin damage. The use of invasive arterial blood pressure monitoring was employed to minimise the risk of soft tissue damage or iatrogenic fractures caused by repeated inflation of standard blood pressure cuffs during the peri-operative period.

On arrival in the anaesthetic room, the patient positioned himself on the operating table with minimal intervention from staff to ensure he was comfortable and reduce the risk of fractures. Two peripheral venous cannulae were sited, as well as an ultrasound-guided radial arterial line, before intravenous induction of anaesthesia with 1mg midazolam, 100mcg fentanyl, propofol by target-controlled infusion, and 50mg rocuronium. Indirect laryngoscopy with video-laryngoscopy revealed a grade 1 view and a 6.5-mm endotracheal tube was placed with a bougie to minimise cervical spine movement. Anaesthesia was maintained by target-controlled infusion of propofol (effective site concentration 4.2μg/ml) and a small bolus of rocuronium which achieved a bispectral index of 32–44. A pressure-controlled ventilation mode was used to deliver pressure of 12-cm H_2_0 on top of a positive end-expiratory pressure (PEEP) of 6cm H_2_0 and achieved tidal volumes of 240 ml. Ventilation parameters were based on his overnight BiPAP settings. Temperature was checked every 30 min and he remained normothermic throughout. Analgesia provided included weight adjusted doses of paracetamol, fentanyl, clonidine and ketorolac, and local infiltration of levobupivacaine. The patient then underwent laparoscopic cholecystectomy with modified positioning of abdominal ports to accommodate the patient′s size, shape, and encountered anatomy. The umbilical port was inserted using an open technique and all others were placed under direct vision. Initial insufflation of the abdomen was to a pressure of 7 mmHg followed by very gradual increase to a maximum abdominal pressure of 12 mmHg, as guided by ventilation parameters. Not unexpectedly, the patient’s pronounced kyphoscoliosis resulted in a challenging variation of intra-abdominal anatomy. The gallbladder was identified with the hepato-cystic triangle positioned to the left of the falciform ligament. Successful completion of surgery took around 2 h. The patient was then electively transferred to the ICU for ongoing level three care. He was nursed on the operating bed until he was awake and adequate neuromuscular reversal had occurred. Arterial blood gases were monitored prior to extubating to ensure a return to baseline (Table 1). Extubation occurred three-hours post-procedure.

**Table 1:** Baseline, intra-operative, and post-operative arterial blood gases

	Baseline Venous Blood Gas	10:58 (start of surgery)	12:42 (end of surgery)	16:58 (post-extubation)	22:12 (evening BiPAP)
PaO_2_ kPa (11.1–14.4)	N/A	28.5	27.6	10.1	11.0
PaCO_2_ kPa (4.7–6.4)	6.10	5.4	6.6	6.4	5.2
pH (7.35–7.45)	7.40	7.45	7.38	7.39	7.43
BE (mmol/L) (-2 – 3)	4.1	4.1	2.9	3.3	1.3

The reference ranges for each analyte are indicated in brackets in the first column. The time of each measurement in relation to the surgical procedure is indicated on the top row

*PaO*_*2*_ partial pressure of oxygen in arterial blood, *PaCO*_*2*_ partial pressure of carbo dioxide in arterial blood, kPa Kilopascals; BE Base excess; *N/A* not available; *BiPAP* Bilevel-positive airway pressure

No specific dietary measures were advised postoperatively although the patient was prescribed laxatives to reduce the risk of constipation associated with analgesia. The patient resumed a normal diet following surgery with no significant issues or the need for additional medication.

The patient was discharged home 48-h post-procedure and was well at six-week follow-up. His symptoms of abdominal pain were reversed by surgery and he remains well on follow-up.

## Discussion

There are very few reports of non-orthopedic surgery having been undertaken in people with severe osteogenesis imperfecta. A literature search identified only three instances, all of which were done laparoscopically; one for a ruptured appendix [5], another for a tubo-ovarian abscess [6], and a third for bariatric surgery [7]. Guidelines for orthopaedic surgery in OI emphasise that decisions around any surgical procedure should involve the patient and family, coupled with a multidisciplinary team [8, 9]. The challenges of anaesthetic management of individuals with severe OI undergoing orthopaedic surgery have also been published [10, 11].

In this case, there was extensive discussion with co-author JA (the patient) and all members of the care team before the surgery in view of the potential risks involved. The decision was made to go ahead with surgery as that the patient’s quality of life was severely impaired as the result of the recurrent episodes of abdominal pain and it was felt that on balance, the benefits outweighed the risk. Many of the anaesthetic considerations when managing a patient with OI will be pertinent irrespective of the patient’s age or the type of surgery being conducted. There is potential for difficult airway management, risk of fractures when positioning (including cervical spine injury during airway manipulation), and with the use of suxamethonium and non-invasive blood pressure cuffs [12]. Sites for intravenous access may be limited due to fractures and musculoskeletal deformity. The risk of hypermetabolic hyperthermia and sensitivity to neuromuscular blocking drugs has been reported to be increased in OI although this is mainly based on case reports [13] and one case series of 49 OI patients undergoing surgery found no evidence to suggest that the risk of hypermetabolic hyperthermia was increased [14]. In this case, there was no evidence of hyperthermia.

Traditional teaching also advises avoiding suxamethonium in OI because of concerns regarding an acute hyperkalaemic response and contraction-induced fractures [15]. These complications have been reported in patients with various neuromuscular diseases and immobilisation [16]. This was the reason that we used rocuronium as a muscle relaxant. Although the patient had had residual neuromuscular blockade several hours postoperatively, this was successfully reversed with sugammadex.

The patient was treated with laparoscopic surgery. This now represents the standard approach for cholecystectomy in the UK, offering a number of advantages over open surgery, including reduced post-operative pain and enhanced recovery. The main anaesthetic concern in laparoscopic surgery is the presence of pneumoperitoneum and its impact on the cardio-respiratory and musculoskeletal systems; this additionally complicates the management of anaesthesia in a patient with OI. The raised intra-abdominal pressure associated with pneumoperitoneum inhibits effective ventilation by increasing pressure under the diaphragm and simultaneously increasing intra-thoracic pressure; this could result in lung barotrauma, rib fractures and development of pneumothoraxes. Ventilation is also impaired by systemic absorption of carbon dioxide, the gas used for insufflation of the abdominal cavity. An intra-abdominal pressure of 12 mmHg was achieved cautiously and allowed adequate surgical access with carbon dioxide controlled at near normal levels for this patient. Ventilation probably benefitted from the reverse Trendelenburg position used in this case. Intercostal chest drain insertion was discussed and prepared for but was not required.

There was a chance that the laparoscopic approach described would not be possible in this case due to the patient’s body habitus, inability to lie flat, and the extremely short distance between xiphisternum and umbilicus, all of which would restrict access to the peritoneum. Pre-operative multidisciplinary discussion focussed heavily on this as well as  the risks associated with open surgery. Conversion to open cholecystectomy was not felt to be in the patient’s best interests due to the increased risks of a larger incision; rib retraction; difficult to manage post-operative pain; and likelihood of prolonged ventilation and critical care stay. The patient was in complete agreement with the multidisciplinary team that if pneumoperitoneum could not be achieved safely the procedure would be abandoned.

Intubation is considered the gold standard for airway protection with pneumoperitoneum as it allows tight control of ventilation parameters in patients with pre-existing respiratory impairment, as was present in this case. Caution during airway manipulation is required in all patients with OI to avoid iatrogenic injury, fracture, or cervical subluxation. Fortunately, pre-operative airway assessment was reassuring in this patient. Despite this, close attention to detail, manual inline stabilisation, and video-laryngoscopy were used to minimise the risk of harm.

Performing elective non-orthopaedic surgery when a patient has an acute fracture carries an increased risk of fracture displacement, haemorrhage, venous thromboembolism, and post-operative complications. Patients with OI have a significant fracture burden and may not be fracture free for any length of time making timing of elective surgery challenging. Accepting that there was unlikely to be an optimal time to operate, the multidisciplinary team took precautions to avoid iatrogenic injury and minimise the risk of fracture in the peri-operative period.

Elective admission to intensive care postoperatively was arranged to facilitate gradual weaning from the ventilator. There were concerns that the patient may become ventilator dependent postoperatively and require tracheostomy formation and long-term ventilation. Again, the impact of this was discussed extensively preoperatively with the patient and also the regional long-term ventilation team and paediatric intensive care specialists who have experience with OI patients. Cardio-pulmonary resuscitation (CPR) was discussed with the patient as part of the anticipatory care plan; if required this would be extremely challenging due to the patient’s body habitus, anatomy, and predisposition to fractures; however, the patient very clearly expressed his wish to receive CPR. A patient advocate was nominated by the patient should he lack capacity to make decisions during his intensive care admission particularly on life altering interventions.

This case report highlights some of the anaesthetic, surgical and critical care considerations for patients with severe OI but illustrates that with careful planning, laparoscopic surgery can be successfully performed in people with severe OI with a favourable outcome.

## References

[1] Hald JD, Langdahl B, Folkestad L, Wekre LL, Johnson R, Nagamani SCS, Raggio C, Ralston SH, Semler O, Tosi L, Orwoll E (2024) Osteogenesis imperfecta: skeletal and non-skeletal challenges in adulthood. Calcif Tissue Int. 10.1007/s00223-024-01236-x 38836890 10.1007/s00223-024-01236-x PMC11606788

[2] Jovanovic M, Marini JC (2024) Update on the genetics of osteogenesis imperfecta. Calcif Tissue Int. 10.1007/s00223-024-01266-5 39127989 10.1007/s00223-024-01266-5 PMC11607015

[3] Sillence DO (2024) A dyadic nosology for osteogenesis imperfecta and bone fragility syndromes 2024. Calcif Tissue Int. 10.1007/s00223-024-01248-7 38942908 10.1007/s00223-024-01248-7 PMC11607092

[4] Folkestad L, Hald JD, Gram J, Langdahl BL, Hermann AP, Diederichsen AC, Abrahamsen B, Brixen K (2016) Cardiovascular disease in patients with osteogenesis imperfect—a nationwide, register-based cohort study. Int J Cardiol 225:250–257. 10.1016/j.ijcard.2016.09.107 27741483 10.1016/j.ijcard.2016.09.107

[5] Farid MI, Baz A, Hemdan MEE, Abdelhamid T, Ebrahim D, Fayed F, Abdel-Haleem E (2024) A successful laparoscopic appendectomy for an adult male patient with osteogenesis imperfecta. Eur J Case Rep Intern Med 11:004738. 10.12890/2024_004738 39247237 10.12890/2024_004738 PMC11379103

[6] Uda T, Masataka K, Ohara Y, Yamakazi H, Ishizuka Y, Ihira K, Tanaka R, Watari H (2018) Laparoscopic surgery in osteogenesis imperfecta (OI) patient with tubo-ovarian abscess (TOA): a case report. Japanese J Gynecol Obstetric Endos 38:128–133. 10.5180/jsgoe.34.1_128

[7] Zani A, Ford-Adams M, Ratcliff M, Bevan D, Inge TH, Desai A (2017) Weight loss surgery improves quality of life in pediatric patients with osteogenesis imperfecta. Surg Obes Relat Dis 13:41–44. 10.1016/j.soard.2015.11.029 26948942 10.1016/j.soard.2015.11.029 PMC5965274

[8] Sakkers RJ, Montpetit K, Tsimicalis A, Wirth T, Verhoef M, Hamdy R, Ouellet JA, Castelein RM, Damas C, Janus GJ, Nijhuis WH, Panzeri L, Paveri S, Mekking D, Thorstad K, Kruse RW (2021) A roadmap to surgery in osteogenesis imperfecta: results of an international collaboration of patient organizations and interdisciplinary care teams. Acta Orthop 92:608–614. 10.1080/17453674.2021.1941628 34180749 10.1080/17453674.2021.1941628 PMC8519518

[9] Bizot P (2024) Orthopedic surgery in osteogenesis imperfecta in adults. Calcif Tissue Int. 10.1007/s00223-024-01306-0 39550451 10.1007/s00223-024-01306-0

[10] Gupta D, Purohit A (2016) Anesthetic management in a patient with osteogenesis imperfecta for rush nail removal in femur. Anesth Essays Res 10:677–679. 10.4103/0259-1162.184612 27746572 10.4103/0259-1162.184612 PMC5062238

[11] Wang H, Huang X, Wu A, Li Q (2021) Management of anesthesia in a patient with osteogenesis imperfecta and multiple fractures: a case report and review of the literature. J Int Med Res 49:3000605211028420. 10.1177/03000605211028420 34190615 10.1177/03000605211028420 PMC8258760

[12] Ross KE, Gibian JT, Crockett CJ, Martus JE (2020) Perioperative considerations in osteogenesis imperfecta: a 20-year experience with the use of blood pressure cuffs, arterial lines, and tourniquets. Children (Basel) 7:214 33171948 10.3390/children7110214 PMC7694628

[13] Porsborg P, Astrup G, Bendixen D, Lund AM, Ording H (1996) Osteogenesis imperfecta and malignant hyperthermia. Is there a relationship? Anaesthesia 51:863–865. 10.1111/j.1365-2044.1996.tb12619.x 8882252 10.1111/j.1365-2044.1996.tb12619.x

[14] Bojanic K, Kivela JE, Gurrieri C, Deutsch E, Flick R, Sprung J, Weingarten TN (2011) Perioperative course and intraoperative temperatures in patients with osteogenesis imperfecta. Eur J Anaesthesiol 28:370–375. 10.1097/EJA.0b013e3283459616 21423018 10.1097/EJA.0b013e3283459616

[15] Chan E, DeVile C, Ratnamma VS (2023) Osteogenesis imperfecta. BJA Educ 23:182–188. 10.1016/j.bjae.2023.01.005 37124171 10.1016/j.bjae.2023.01.005 PMC10140476

[16] Azar I (1984) The response of patients with neuromuscular disorders to muscle relaxants: a review. Anesthesiology 61:173–187. 10.1097/00000542-198408000-00011 6087697 10.1097/00000542-198408000-00011

